# 

**Published:** 1908-04-04

**Authors:** 


Telegraphic Address: Ospedale, London.

THE MODERN MEDICAL JOURNAL.

Telephone Number: 2734 Gerrard.

NEW SERIES. No. 55, Vol. III.

No. 1127. Vol. XLIV.

SATURDAY, APRIL 4TH, 1908.

PRICE THREEPENCE.

